# The D Gene in CDR H3 Determines a Public Class of Human Antibodies to SARS-CoV-2

**DOI:** 10.3390/vaccines12050467

**Published:** 2024-04-27

**Authors:** Meng Yuan, Ian A. Wilson

**Affiliations:** 1 Department of Integrative Structural and Computational Biology, The Scripps Research Institute, La Jolla, CA 92037, USA; wilson@scripps.edu; 2 The Skaggs Institute for Chemical Biology, The Scripps Research Institute, La Jolla, CA 92037, USA

**Keywords:** SARS-CoV-2, broadly neutralizing antibody, public antibody, D gene, CDR H3

## Abstract

Public antibody responses have been found against many infectious agents. Structural convergence of public antibodies is usually determined by immunoglobulin V genes. Recently, a human antibody public class against SARS-CoV-2 was reported, where the D gene (IGHD3-22) encodes a common YYDxxG motif in heavy-chain complementarity-determining region 3 (CDR H3), which determines specificity for the receptor-binding domain (RBD). In this review, we discuss the isolation, structural characterization, and genetic analyses of this class of antibodies, which have been isolated from various cohorts of COVID-19 convalescents and vaccinees. All eleven YYDxxG antibodies with available structures target the SARS-CoV-2 RBD in a similar binding mode, where the CDR H3 dominates the interaction with antigen. The antibodies target a conserved site on the RBD that does not overlap with the receptor-binding site, but their particular angle of approach results in direct steric hindrance to receptor binding, which enables both neutralization potency and breadth. We also review the properties of CDR H3-dominant antibodies that target other human viruses. Overall, unlike most public antibodies, which are identified by their V gene usage, this newly discovered public class of YYDxxG antibodies is dominated by a D-gene-encoded motif and uncovers further opportunities for germline-targeting vaccine design.

## 1. SARS-CoV-2 Escapes from Major Classes of Neutralizing Antibodies

The severe acute respiratory syndrome coronavirus 2 (SARS-CoV-2) spike protein is a trimeric type I fusion machine that facilitates viral entry. The spike protein is also the main target for neutralizing antibodies. Most of the approved coronavirus disease 2019 (COVID-19) vaccines only deliver the spike protein [1]. We and others have reported several public antibody classes elicited by the ancestral viral infection or vaccination against SARS-CoV-2, e.g., public antibodies encoded by IGHV3-53/3-66 [2,3] and IGHV1-58/IGKV3-20 [4]. These antibodies usually target the receptor-binding site (RBS) of the SARS-CoV-2 spike protein, compete with receptor binding, and possess high neutralization potency. Additionally, IGHV1-24 antibodies have been frequently found to target the ‘supersite’ in the N-terminal domain (NTD) of the SARS-CoV-2 spike, exhibiting strong neutralization potency [5,6,7]. IGHV1-46/IGKV3-20 encode a prevalent public clonotype of broadly neutralizing antibodies targeting the S2 stem region of betacoronaviruses [8,9]. These public antibodies are frequently elicited upon infection or vaccination and contribute substantially to protection against viral infection or disease severity.

**Figure 1:**
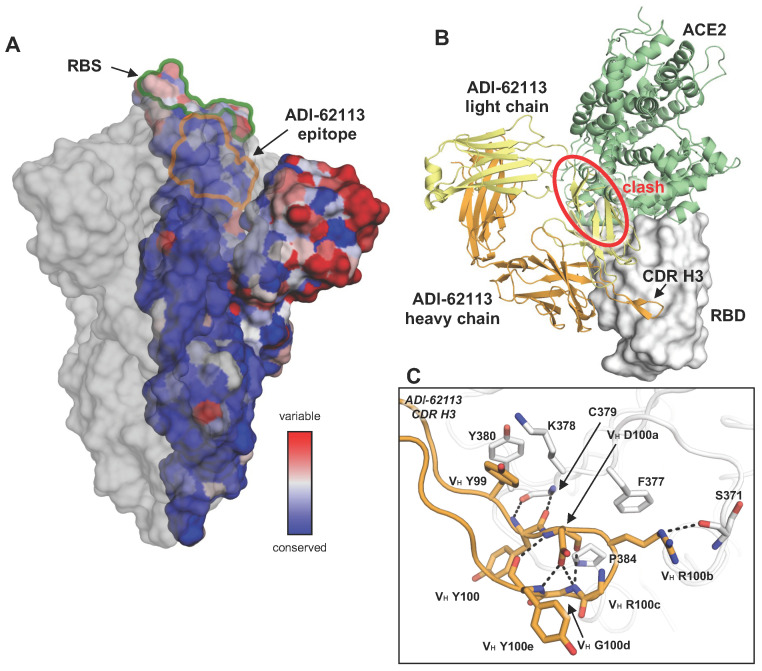
ADI-62113 as a model for YYDxxG antibodies. (**A**) Conservation of the spike protein across SARS-CoV-2 variants (PDB 7SXZ). One protomer with RBD (receptor-binding domain) in its up conformation shows conservation, as calculated by ConSurf [14] and color-coded here. The ADI-62113 epitope is defined by its buried surface area (BSA > 0 Å^2^) on the RBD, as calculated by Proteins, Interfaces, Structures and Assemblies (PISA; www.ebi.ac.uk/pdbe/prot_int/pistart.html, accessed on 1 July 2023) and outlined. The other two protomers of the spike trimer are shown as a transparent grey surface. (**B**) The SARS-CoV-2 RBD is shown as a white surface, while the heavy and light chains of ADI-62113 are in orange and yellow, respectively (PDB 7T7B). The RBD–antibody structure is superimposed onto an RBD-ACE2 (angiotensin-converting enzyme 2) structure (PDB 6M0J) and illustrates that ADI-62113 would clash (red circle) with ACE2 (green). (**C**) Detailed interactions between the CDR H3 (heavy-chain complementarity-determining region 3) YYDxxG motif of ADI-62113 with SARS-CoV-2 RBD. Hydrogen bonds are indicated by dashed lines.

Not long after the beginning of the COVID-19 pandemic, a number of breakthrough viruses (variants of concern) started to emerge. Further evolution of SARS-CoV-2 variants, especially Omicron and its subvariants, was found to enhance transmissibility [10]. The heavily mutated spike protein also results in evasion of established human immune responses against the wildtype and subsequent variants [11,12]. Plasma elicited by previous SARS-CoV-2 infection or vaccination exhibit reduced neutralization activity against Omicron and its subvariants. All of the antibodies under emergency use authorization by the US FDA have now been escaped by these variants [13]. Moreover, all of the above-mentioned anti-RBD antibody public classes, targeting the RBS, one of the most variable regions of the spike protein (Figure 1A), were evaded by at least one Omicron subvariant, while non-RBD broadly neutralizing antibodies usually exhibit modest neutralization potency. Therefore, identifying potent neutralizing antibodies targeting conserved sites on the spike protein is essential for universal vaccine design against SARS-CoV-2.

## 2. Recurring YYDxxG Motif in CDR H3 Targets a Conserved Site on SARS-CoV-2 Spike

**Figure 2:**
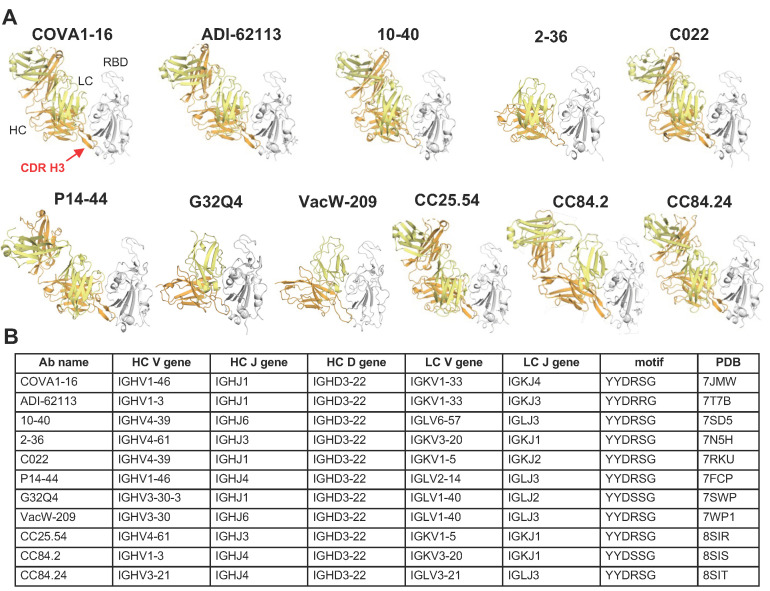
Structural comparison of YYDxxG antibodies. (**A**) All YYDxxG antibodies target SARS-CoV-2 RBD in a similar binding mode. The CDRs H3 (indicated by a red arrow in the first structure COVA1-16 as a representative) are responsible for the majority of the antigen interactions. The SARS-CoV-2 RBD is shown in whitish grey, while the heavy and light chains of the antibodies are in orange and yellow. For antibodies 2-36, VacW-209, and G32Q4, only the variable domain is available in the published structure. (**B**) Germline genes of the YYDxxG antibodies with available structures.

Tens of thousands of different monoclonal antibodies (mAbs) have been isolated from COVID-19 convalescents and vaccinees. The functional, genetic, and structural features of many of these mAbs have been characterized. We and others discovered that a recurring antibody response was due to a ‘YYDRxG’ motif on the heavy-chain complementarity-determining region 3 (CDR H3) [15,16,17] and primarily responsible for its binding to a relatively conserved site on the RBD of SARS-CoV-2 spike. This motif is somatically hypermutated from an IGHD3-22-encoded YYDSSG fragment. Both unmutated and convergently mutated YYDRxG-containing antibodies have been identified in SARS-CoV-2 RBD antibodies [18,19]; the motif is therefore referred as ‘YYDxxG’ motif here as some limited variability has been seen at the R position (Figure 2). The structures of these antibodies, COVA1-16, ADI-62113, and 10-40, in complex with SARS-CoV-2 spike protein [15,16,17] showed that all of the YYDxxG antibodies convergently targeted a conserved site on the SARS-CoV-2 spike (Figure 1A). This site does not overlap with the RBS, and thus other antibodies targeting this site (e.g., CR3022, S2A4) usually do not sterically hinder the receptor binding and exhibit no or minimal neutralization activity [20,21]. However, the YYDxxG antibody class is an exception to the notion that, in many cases, antibody neutralization potency and breadth are usually mutually exclusive [22]; here targeting a conserved epitope confers neutralization breadth while the particular approach angle of YYDxxG antibodies facilitates direct steric hindrance to receptor binding and thereby confers high neutralization potency (Figure 1B). 

The structural convergence of most public antibodies is determined by their common immunoglobulin V genes, especially for the germline-encoded CDRs 1 and 2, but CDR H3 has also been considered in this designation [23]. Some well-known examples include the germline-encoded antibody classes to SARS-CoV-2, such as those encoded by IGHV3-53/3-66 [2,3], as well as anti-HIV VRC01-like antibodies encoded by IGHV1-2 [24] and anti-influenza hemagglutinin (HA) stem antibodies encoded by IGHV1-69 [25]. However, the analysis here shows that the public response of this recently discovered class of antibodies is determined by its D gene, which can encode a YYDSSG motif. Here, we analyzed the sequences of over 11,000 antibodies isolated from COVID-19 patients and mRNA vaccinees from mAbs reported in CoV-AbDab [26], Wang et al. [27], and the NCBI GenBank database [28]. Notably, such antibodies have been frequently found in COVID-19 convalescents and vaccinees during the past three years. We found 110 mAbs (~1%) containing the motif with a convergent mutation, i.e., the YYDRxG motif, and all of the identifiable D regions, are exclusively encoded by IGHD3-22, while the V and J genes vary [15]. Current structures show that R or S can follow YYD (Figure 2) and, hence, we generalize it here as the YYDxxG motif (the underlined x refers to V_H_ S100b or R100b; Kabat numbering is used throughout this paper), where the germline S can be converted to an R by a single base change. The RBD can accommodate V_H_ S100b, while a somatic hypermutation to V_H_ R100b confers additional interactions with RBD-S371 and F377 (Figure 1C). The mutation of serine (AGT in the IGHD3-22 germline) requires only one nucleotide change to arginine (AGA or AGG). 

The CDR H3 is responsible for the vast majority of antigen interactions in this class of antibodies. For both COVA1-16 and ADI-62113, approximately 70% of the total buried surface area (BSA) on the RBD is formed by CDR H3 [15,16]. Detailed structural analyses illuminate how the _99_YYDxxG_100d_ motif interacts with a highly conserved site on SARS-CoV-2 RBD and determines the public class. The _99_YY_100_ dipeptide forms hydrophobic interactions with the RBD core, and the subsequent _100a_DxxG_100d_ forms a type 1 β-turn, stabilized by a hydrogen bond between the carboxyl oxygen of V_H_ D100a and the backboneamides of V_H_ G100d and Y100e; the carbonyl oxygen of V_H_ Y100e also hydrogen bonds with the backbone amide of V_H_ D100a (Figure 1C). V_H_ R100b is located at the β-turn tip of CDR H3, where its aliphatic moiety interacts with the aromatic ring of RBD-F377, and its guanidinium group hydrogen bonds with RBD-S371 (Figure 1C). Furthermore, V_H_ G100d with a positive phi (φ) angle allows for the displacement of the inserted V_H_ Y100e in the descending G1 β-bulge, thus creating space for V_H_ Y100 to interact with the RBD.

## 3. The Angle of Approach of YYDxxG Antibodies Facilitates IgG Bivalent Binding and Avidity Effects

**Figure 3:**
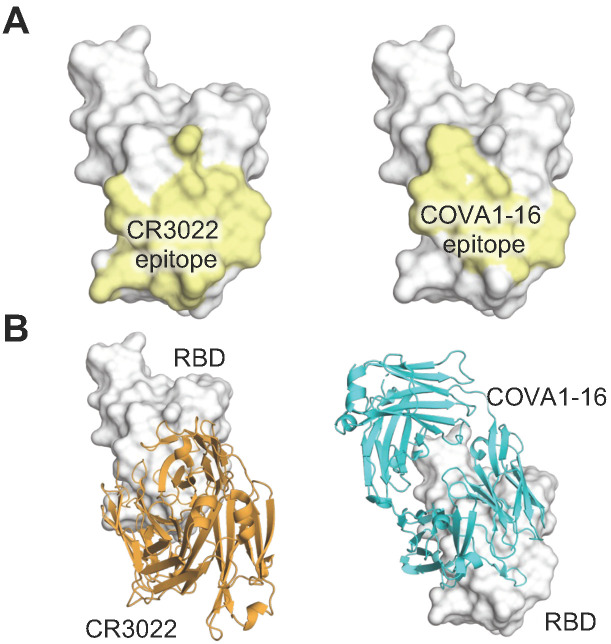
CR3022 and COVA1-16 target a similar epitope but with distinct angles of approach. (**A**) Epitopes and (**B**) structures of CR3022 (orange, PDB 6W41) and COVA1-16 (cyan, PDB 7JMW) in complex with the RBD (whitish grey) are depicted here. The epitopes are defined by the buried surface area (BSA > 0 Å^2^) on the RBD when bound to the antibody.

Avidity can be an important factor in IgG binding due to its bivalent nature. Most anti-HIV antibodies lack any avidity effect due to steric restrictions and low spike density that preclude bivalent binding within and between spikes [29,30]. Likewise, CR3022, a mAb previously isolated from a SARS convalescent that binds to a cryptic conserved site of sarbecoviruses including SARS-CoV-2 [20] (Figure 3), exhibited a minimal avidity effect, where IgG did not confer much stronger activity compared to its monovalent Fab counterpart (increased by only 1.4 fold) [31]. In contrast, the YYDxxG antibody COVA1-16 binds to a very similar epitope but exhibited a strong avidity effect, where the IgG exhibited >1000-fold activity compared to its Fab [16]. The drastic difference between the avidity effects of CR3022 and COVA1-16 may be attributed to the different angles of approach targeting the spike protein (Figure 3). The YYDxxG antibody approaches the RBD from a downward angle (relative to an upright spike embedded in a membrane), which may facilitate an inter-spike bridging interaction [16]. In other words, changing the angle of approach of antibodies to an epitope may facilitate bivalent binding and therefore avidity, which can enhance the potency of the IgG. 

## 4. YYDxxG Antibodies Are Frequently Found in COVID-19 Convalescents and Vaccinees

At the beginning of the pandemic, our colleagues isolated COVA1-16 [16] from a COVID-19 convalescent and we determined its crystal structure. This antibody contains a 20-amino acid long CDR H3 that contains the YYDxxG motif encoded by IGHD3-22. The CDR H3 occupies approximately 70% of the surface area of the antigen buried by the antibody, suggesting that CDR H3 dominates the recognition by antibody. YYDxxG antibodies have been frequently found in COVID-19 convalescents during the past three years. For example, Wang et al. screened mAbs isolated from COVID-19-convalescent patients, and found one mAb, 2-36, that exhibited cross-neutralizing activity against SARS-CoV-1 [32]. Jette et al. investigated a class-4 human antibody C022 that was previously isolated from a COVID-19 donor [4], which was cross-reactive with a broad range of sarbecoviruses and exhibited a strong avidity effect [33]. Zou et al. demonstrated a YYDxxG antibody, P14-44, also isolated from a COVID-19 convalescent, neutralized a broad range of SARS-CoV-2 variants, as well as other sarbecoviruses, and was highly resistant to mutations [34]. Since November 2021, Omicron variants emerged and quickly spread throughout the world. Omicron is highly resistant to most neutralizing antibodies. However, YYDxxG antibodies, including COVA1-16 [35], C022 [36], ADI-62113 [15], 10-40 [17], and G32Q4 [37], retained neutralization activity against Omicron BA.1, albeit with a reduction in activity. 

In addition to COVID-19 convalescent patients [15,16], YYDxxG motif-containing antibodies have also been found in vaccinees [38,39]. Ju et al. isolated an antibody VacW-209 from inactivated vaccine-elicited PBMCs that also exhibited neutralization activity against Omicron BA.1 [40]. Recently, we and colleagues reported on antibodies from individuals who were infected with SARS-CoV-2 and then received an mRNA vaccine [41]. Antibodies were selected with broad reactivity against Omicron and other sarbecoviruses, and clustered into two major groups by antibody competition experiments. Notably, group 1 bnAbs were strongly enriched for the IGHD3-22-encoded CDR H3 YYDxxG motif and possessed long CDR H3 loops, while their V and J genes varied (Figure 2B). In contrast, none of the group 2 bnAbs were encoded by IGHD3-22 or contained the YYDxxG motif. Some YYDxxG antibodies, including CC25.54, CC84.2, and CC84.24, exhibit extraordinary neutralization breadth against many recent variants, including BA.1, BA.2, BQ.1.1, and XBB.1.5, with reduced neutralization compared to the prototype SARS-CoV-2 [42]. The reduced neutralizing activity and the binding to spike [42] may be due to the more closed form of the pre-fusion spike of Omicron and subvariants [43,44], thereby resulting in less exposure of the cryptic epitope that is targeted by the YYDxxG antibodies.

Besides their surprisingly high frequency, YYDxxG antibodies also present some other interesting features. First, YYDxxG antibodies are structurally convergent (Figure 2A), where the antibodies bind to a nearly identical epitope with a highly similar angle of approach, and CDR H3 dominates the interaction. Second, all of the YYDxxG antibodies are encoded by the same D gene, i.e., IGHD3-22 (Figure 2B), but the VH/JH and VL/JL genes vary, indicating that the D-gene determines the public response. 

Through targeting a conserved site, all of the YYDxxG antibodies described here exhibit cross reactivity to sarbecoviruses, including SARS-CoV-2 and SARS-CoV-1 [15,16,17,32,33,34,37,40,41,42]. Moreover, most of these antibodies preserve their binding and neutralization against SARS-CoV-2 variants with strong escape mutations, including Omicron BA.1, although binding and neutralizing activity to BA.2 and subsequent subvariants were reduced [42]. Nevertheless, their overall neutralization activity highlights the therapeutic potential of this class of broadly neutralizing antibodies.

## 5. Insights and Potential Applications of CDR H3-Dominant Public Antibodies

The public class of YYDxxG antibodies is intriguing and warrants attention due to three key features. First, unlike most public antibodies that share V genes, this D gene encodes a YYDxxG motif in CDR H3 that forms extensive and critical interactions with the antigen and determines the structural convergence of these antibodies with a variety of V and J genes. It is interesting to investigate other antibody classes dominated by D genes. We previously observed a similar situation where the D3-9 encoded region dominates antibody interactions with the influenza A HA stem [45]. Distinct from the same reading frame being used to encode the YYDxxG motif, the D gene that encodes the anti-influenza antibodies can be read in two different reading frames to engage the HA [45]. Using the first reading frame, the D gene encodes a ‘YFDWL’ motif that is used in the 21- and 20-residue CDRs H3 of S9-3-37 (YFDWL) and F16v3 (YFEWL), which dominates the antibody–antigen interaction with 83% and 71% of the total BSA [45,46] (Table 1). On the other hand, antibody 31.b.09 uses the second reading frame of IGHD3-9 that encodes ‘ILTG’ in CDR H3, which accounts for approximately 30% of the total BSA [47]. It is reasonable to surmise that D gene-determined public classes may be better targets for germline-targeting vaccine design, as D genes encode much fewer amino acids compared to V and J genes and therefore may be easier to elicit in order to achieve high affinity binding with fewer mutations. Second, YYDxxG antibodies target a non-RBS conserved site in the SARS-CoV-2 RBD, but the antibody angle of approach results in steric hindrance of receptor binding and enhances neutralization potency and breadth. This finding is similar to our previous study, where antibodies that span the RBS-D/CR3022 sites take advantage of the properties of both sites [22]. Third, YYDxxG antibodies target a similar epitope to that of a non-YYDxxG antibody, CR3022, but with a distinct angle of approach and some variations in the epitopes at the periphery (Figure 3). In contrast to the CR3022 IgG, YYDxxG antibodies exhibit a strong avidity effect (i.e., bivalent IgGs display much higher activity than their corresponding monovalent Fabs), highlighting that the precise approach angle of mAbs can play a vital role in their avidity. 

The epitope of the YYDxxG antibodies is generally conserved, but Omicron and its subvariants do exhibit a few mutations that reduce the binding and neutralization of this class of antibodies. In vivo and in vitro affinity maturation may increase the neutralization potency and breadth of these antibodies, which could lead to high therapeutic potential against SARS-like viruses. In addition to YYDxxG antibodies, we and colleagues previously described another two public classes of neutralizing antibodies targeting a conserved epitope [9]. These antibodies are encoded by IGHV1-46/IGLV2-14 and IGHV1-46/IGKV3-20. Both sets of antibodies target the conserved S2 stem region across betacoronaviruses [9]. These public antibodies could suggest historical imprinting of our immune system against coronaviruses. Importantly, structural insights of the conserved regions targeted by public antibodies have potential for germline-targeting design of more universal vaccines against coronaviruses. 

## 6. CDRH3-Dominant Antibodies Are Found against Various Viruses

**Figure 4:**
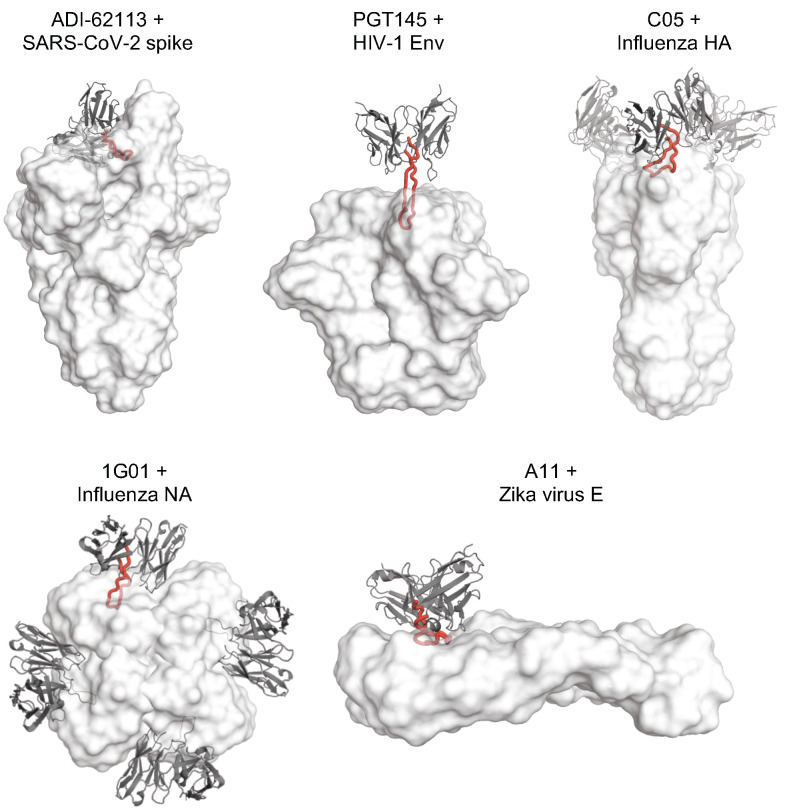
Representative antibodies with a dominant CDR H3 in complex with viral surface proteins. Variable domains of antibodies are represented by grey cartoon, and viral antigens by white surface. CDRs H3 are shown in red tubes. Only one CDR H3 is shown in red if multiple antibodies are bound in a viral protein.

The CDR H3 of YYDxxG antibodies dominates the interactions with SARS-CoV-2 spike protein, where approximately 70% of the total BSA on the antigen is formed by interaction with CDR H3 of COVA1-16 and ADI-62113 [15,16]. In addition to SARS-CoV-2, CDRH3-dominant antibodies have been found against other viruses (Table 1 and Figure 4). 

The CDR H3 of anti-HIV mAbs are generally considerably longer on average than those found in the naïve repertoire [49]. In particular, the CDR H3 of most bnAbs targeting the V2-apex site of HIV-1 envelop glycoprotein (Env) are not only exceptionally long (≥24 and up to 37 residues) [50,51], but also usually dominate the antibody–antigen interactions (surface area buried by CDR H3 > 70% of the total BSA of the antibody). The HIV-1 apex, comprised of V1 and V2 loops, is a well-documented target of bnAbs, such as PGT145, PG9, CAP256-VRC26.25, etc. [51,52,53,54]. In fact, antibodies targeting the V2 apex of the HIV-1 Env trimer comprise one of the most commonly elicited categories of bnAbs [52]. These antibodies usually use their ultralong CDR H3 to engage and deeply penetrate into a hole at the center of the apex of the three protomers of the trimer and interact with N-glycans surrounding the apex hole (e.g., N160 glycan). For instance, approximately 80% of the surface area buried by PGT145 is contributed by its ultralong CDR H3 (31 amino acids) [53]. The long anionic CDR H3 of PGT145 penetrates between glycans at the apex of the Env trimer and interacts with amino-acid residues from all three Env protomers [53]. Another V2 apex-targeting bnAb, PG9, also has a CDR H3 that consists of 28 amino acids, which dominates the interaction with the HIV-1 Env trimer (>71% of the total BSA). The extended CDR H3 of PG9 has a hammerhead shape and penetrates the glycan shield that covers the V1/V2 loops on the apex and interacts with a conserved epitope of the Env trimer [54]. A clonally related bnAb, PG16, exhibits a similar binding pose to the antigen with a smaller BSA percentage contributed by CDR H3, which still accounts for more than half of the total interactions [55]. The CDR H3 of both PG9 and PG16 present a hammerhead-like motif, which is different from that of PGT145-like antibodies that adopts a long, anti-parallel β-hairpin-like structure [53] (Figure 3). A lineage of V2-targeting bnAbs (CAP256-VRC26 antibody lineage) was isolated from a donor CAP256 [51]. These antibodies are clonally related, encoded by IGHV3-30 and IGLV1-51 with the CDR H3 of 35–37 residues. Recently, a structure of a potent antibody from this lineage, CAP256-VRC26.25, was determined in complex with an HIV-1 Env trimer [52]. Similar to other V2 apex targeting antibodies, the CDR H3 of CAP256-VRC26.25 also dominates the antibody–antigen interaction and accounts for 87% of the total BSA [52]. The 36-residue CDR H3 of VRC26.25 penetrates the glycan shield, inserts into the apex hole, and recognizes β-strands of the V1V2 region. Landais et al. isolated the PCT64 lineage of bnAbs from Donor PC64, an HIV-1 subtype A-infected individual. These antibodies compose of a PGT145-like lineage that possess a 25-amino acid long CDR H3 and target the HIV Env V2-apex epitope that involves the N160 glycan [56,57]. This antibody lineage exhibits relatively lower somatic hypermutation (SHM) rate (around 10%) and no indels compared to other anti-HIV bnAbs, which makes it an attractive vaccine target. Moreover, bnAbs PGDM1400-1412 [58] and CH01-CH04 [59] also possess a long CDR H3 and target the V2 apex of HIV Env trimers. In addition to being exceptionally long, the CDR H3 of V2-apex-targeting bnAbs have two other common features: they are usually anionic and contain sulfated tyrosines. These features facilitate specific electrostatic interactions with epitope residues in the V2-apex region of HIV-1 Env. 

Another interesting feature of V2-apex-targeting bnAbs is that many lineages use a common germline encoded a YYD motif in their CDR H3 to interact with the epitope [60]. PG9/PG16, the CAP256-VRC26 lineage, and the PCT64 lineage all possess the YYD motif. A single mutagenesis study of the YYD motif showed partial or complete loss of antibody neutralization, highlighting the critical role of this motif [60]. One or two of the tyrosines in the YYD motif are usually sulfated and form extensive interactions with the HIV Env antigen. Note that the YYD motif of anti-HIV antibodies partially resembles the YYDxxG motif of anti-SARS-CoV-2 antibodies reviewed above, but the two are encoded by different D genes (IGHD3-03 and IGHD3-22, respectively). This universality of the motif in antibodies targeting various pathogens highlights the versatility of this tripeptide in CDR H3 for antigen recognition. 

CDRH3-dominant interactions have also been found in anti-influenza bnAbs. In 2012, Ekiert et al. characterized a bnAb called C05, which neutralizes strains from various subtypes of influenza A [61]. C05 is encoded by IGHV3–23, IGKV1–33, and putatively IGHD6-13. The antibody targets the RBS of the HA. The antibody recognition is dominated by its 24-residue CDR H3, which accounts for 81% of the binding surface area and has been described as essentially a one CDR loop binding antibody. The antibody binds near the membrane-distal end of the HA trimer. Its CDR H3 extends far from the antibody surface with the tip forming a β-hairpin and inserts into the HA RBS, therefore inhibiting virus attachment by impeding binding to the host receptor sialic acid. In 2018, McCarthy et al. demonstrated another lineage of anti-influenza bnAbs, called the K03.1-12 lineage, that targets the influenza HA RBS [62]. This lineage of bnAbs is encoded by IGHV1-2, IGHD6-19, and IGLV2-23. The structural determination of a member of this lineage, K03.12, showed that its binding pose is remarkably similar to C05, although they are encoded by different germline genes [62,63]. K03.12 also uses an ultralong CDR H3 (86% of the total antibody BSA) to target the HA RBS. Another bnAb lineage, lineage 652, also targets the HA RBS in a CDR H3 dominant manner, where this CDR accounts for approximately 85% of the total BSA [64]. In 2022, Sun et al. isolated a lineage of bnAbs encoded by IGHV3-48/IGHD2-2/IGKV1-12 that cross-neutralizes influenza A group 1 and group 2 viruses, including H1N1, H3N2, H4N2, and H7N9 [65]. The structures of 28-12, a member of this lineage, demonstrated that the antibody targets the stem region of HA, more specifically, the N-terminal region of HA2 of H3 (fusion peptide and helix A). 28-12 is also in contact with a few residues in HA1 when binding to H1. Antigen recognition by 28-12 is also dominated by its CDR H3, which accounts for approximately 79% of the overall BSA. 

In addition to the HA, the viral surface glycoprotein neuraminidase (NA) is another attractive vulnerable target of nAbs against influenza viruses. 1G04, 1E01, and 1G01 form a three-member clonal family encoded by IGHV3-20 and IGKV1-9 and broadly neutralize influenza viruses that belong to influenza A groups 1 and 2. Their 21-residue CDRs H3 dominate the antibody–antigen interaction by accounting for 67%, 66%, and 77% of the total BSA. The CDR H3 inserts into the recessed active site of the NA and thereby blocks cleavage of host sialic acid that facilitates neutralization [66]. 

Zika and dengue viruses (ZIKV and DENV) belong to the genus *Flavivirus*. The envelope (E) protein is highly conserved among flaviviruses. The flavivirus E protein represents the major target for nAbs. Cross-reactive nAbs have been discovered targeting flavivirus E proteins [67,68,69,70,71,72,73]. A11, an antibody isolated from a DENV-infected patient, exhibits broad and potent neutralization against flaviviruses, including ZIKV and DENV [68,74,75]. A complex structure of A11 and ZIKV E protein demonstrated that the antibody recognizes the antigen mainly through its CDR H3 (83% of total BSA). One-third of the CDR H3 residues form a helix and interacts with the N-glycan at N154 of the E protein. Interestingly, the anti-flavivirus antibody also has a IGHD3-22-encoded YYDxxG motif in its CDR H3 (YYDSTG) [74]. 

Only ~1% of human antibodies in the Abysis database have a CDR H3 of 24 or more residues [76]. Here, we show that antiviral antibodies possessing ultralong CDRs H3 often dominate antibody–antigen interactions. These dominant CDRs, especially those that determine public antibody responses, may have evolutionary implications, where certain D genes or CDR H3 motifs that recognize common viral pathogens have great importance in immediate recognition of viral antigens and thus constitute an SOS response [77].

## 7. Conclusions

Since the emergence of the COVID-19 pandemic, an unprecedented effort has been directed toward discovery of monoclonal neutralizing antibodies against SARS-CoV-2. However, the virus’ rapid evolution has facilitated evasion from all human neutralizing antibodies in emergency use authorization. In this paper, we have reviewed and analyzed the structure and function of a class of broadly neutralizing antibodies that contain a YYDxxG motif in their CDR H3 and where their relatively long and extended CDR H3 dominates the interaction. These antibodies were isolated from various cohorts of COVID-19 convalescents and vaccinees, and they target the SARS-CoV-2 receptor-binding domain in a structurally convergent manner due to a conserved YYDxxG motif. Although targeting a conserved epitope that does not overlap with the receptor-binding site, the particular angle of approach of YYDxxG antibodies facilitates the blocking of receptor binding to the RBD, which enhances their neutralizing potency. Intriguingly, all YYDxxG antibodies are encoded by the same D gene but have a variety of V and J genes, demonstrating a D-gene-specific public class of antibodies. We also illustrate here that CDR H3-dominant antibodies have been widely found against different viruses, including HIV-1, influenza virus, and flaviviruses, suggesting a common strategy that human humoral immune system adopts against various pathogens. Despite the high variation in the sequence and structure of CDR H3 of antibodies elicited against different antigens, investigation of the structural and functional properties of CDR H3 of antiviral antibodies has revealed some common structural features as well as restricted use of germline genes for antibody recognition of human viral pathogens. Germline-targeting is now therefore a widely accepted concept for next-generation vaccine design for public antibodies where the germline VH and VL encodes residues that dominate the antigen recognition [78,79,80,81,82]. Here, we show that public antibodies where the D gene dominates antigen recognition are not rare and may also have great potential for germline-targeting vaccine design.

## Figures and Tables

**Table 1:** CDRH3-dominant antibodies against various viruses.

Antigen	Antibody Category/Epitope	Antibody Name	BSA by CDR H3 (%)	Putative D Gene *	CDR H3 Sequence	CDRH3 Length	PDB
SARS-CoV-2 spike	YYDxxG	COVA1-16	68%	IGHD3-22	PPRNYYDRSGYYQRAEYFQH	20	7JMW
SARS-CoV-2 spike	YYDxxG	ADI-62113	72%	IGHD3-22	AARPYYDRRGYFFRADYFQH	20	7T7B
HIV-1 Env	V1V2 apex	PGT145	80%	IGHD4-17	GSKHRLRDYFLYNEYGPNYEEWGDYLATLDV	31	5V8L
HIV-1 Env	V1V2 apex	PG9	71%	IGHD3-03	EAGGPDYRNGYNYYDFYDGYYNYHYMDV	28	7T77
HIV-1 Env	V1V2 apex	CAP256-VRC26.25	87%	IGHD3-03	DLREDECEEWWSDYYDFGKQLPCAKSRGGLVGIADN	36	6VTT
HIV-1 Env	V1V2 apex	PCT64.LMCA	66%	IGHD3-03	GVETYDFWSGYDDHYYDYYFRDVW	24	7T73
Influenza HA	RBS	C05	81%	IGHD6-13	HMSMQQVVSAGWERADLVGDAFDV	24	4FP8
Influenza HA	RBS	K03.12	86%	IGHD6-19	DLTLMYVFDSGWARGAHDYYGMDV	24	5W08
Influenza HA	RBS	652-I-7-6	85%	IGHD2-2	APPYCTSASCPDDYYYYYMDV	21	6Q0I
Influenza HA	stem	28-12	79%	IGHD2-2	DRGCSSTNCYVVGYYFYGMDV	21	7X6O
Influenza HA	stem	S9-3-37	83%	IGHD3-9	EFRTQIVLGYFDWLEGNAFDM	21	6E3H
Influenza HA	stem	F16v3	71%	IGHD3-9	DSQLRSLLYFEWLSQGYFDY	20	3ZTJ
Influenza NA	catalytic site	1G01	77%	IGHD3-10	TSSWGDYTRGPEPKITWYFDL	21	6Q23
ZIKV E	150 loop	A11	83%	IGHD3-22	DGVRFYYDSTGYYPDSFFKYGMDV	24	5LCV

* Putative D genes are assigned by IMGT/V-QUEST [48] if not provided in the original literature.
